# Editorial: Diversity, function, and application of microbes in the fermentation or production of traditional food

**DOI:** 10.3389/fmicb.2025.1751159

**Published:** 2026-01-06

**Authors:** Weiwei Dong, Shenxi Chen, Yuanliang Hu

**Affiliations:** 1 Hubei Key Laboratory of Edible Wild Plants Conservation and Utilization, College of Life Sciences, Hubei Normal University, Huangshi, China; 2 Hubei Key Laboratory of Quality and Safety of Traditional Chinese Medicine Health Food, Jing Brand Co., Ltd., Daye, Hubei, China

**Keywords:** functional microbes, metabolites biosynthesis, microbial ecology, synthetic microbial consortia, traditional fermented foods

Traditional fermented foods are a living heritage of human culture, embodying terroir and the long-term domestication of microbes. They harbor diverse microbes (for example, lactic acid bacteria, acetic acid bacteria, *Bacillus*, yeasts), which, through interactions and co-metabolism, convert nutrients in raw materials into complex flavor compounds, while enhancing nutrition and safety and conferring potential probiotic functions. With the widespread application of modern microbial ecology tools such as amplicon sequencing, metagenomics, and metabolomics, we can now characterize these intricate communities with unprecedented resolution. This Research Topic assembles 24 papers that span fundamental community ecology, applied strain selection, and innovative fermentation strategies. Overall, they illustrate how a systematic understanding of microbial diversity and function in traditional fermentation is demystifying experience-based craftsmanship and enabling evidence-based application of key functional microbes in food and health.

In this editorial, we synthesize key findings across thematic clusters and outline four major areas (Figure 1): (1) microbial ecology and community dynamics across diverse fermentation systems; (2) functional microbes and the biosynthesis of their metabolites; (3) application-oriented innovations and synthetic strategies; and (4) future perspectives for both research and industry. We highlight how these collective insights push the boundaries of food microbiology and consider their broader applicability across global traditional food systems.

**Figure 1:**
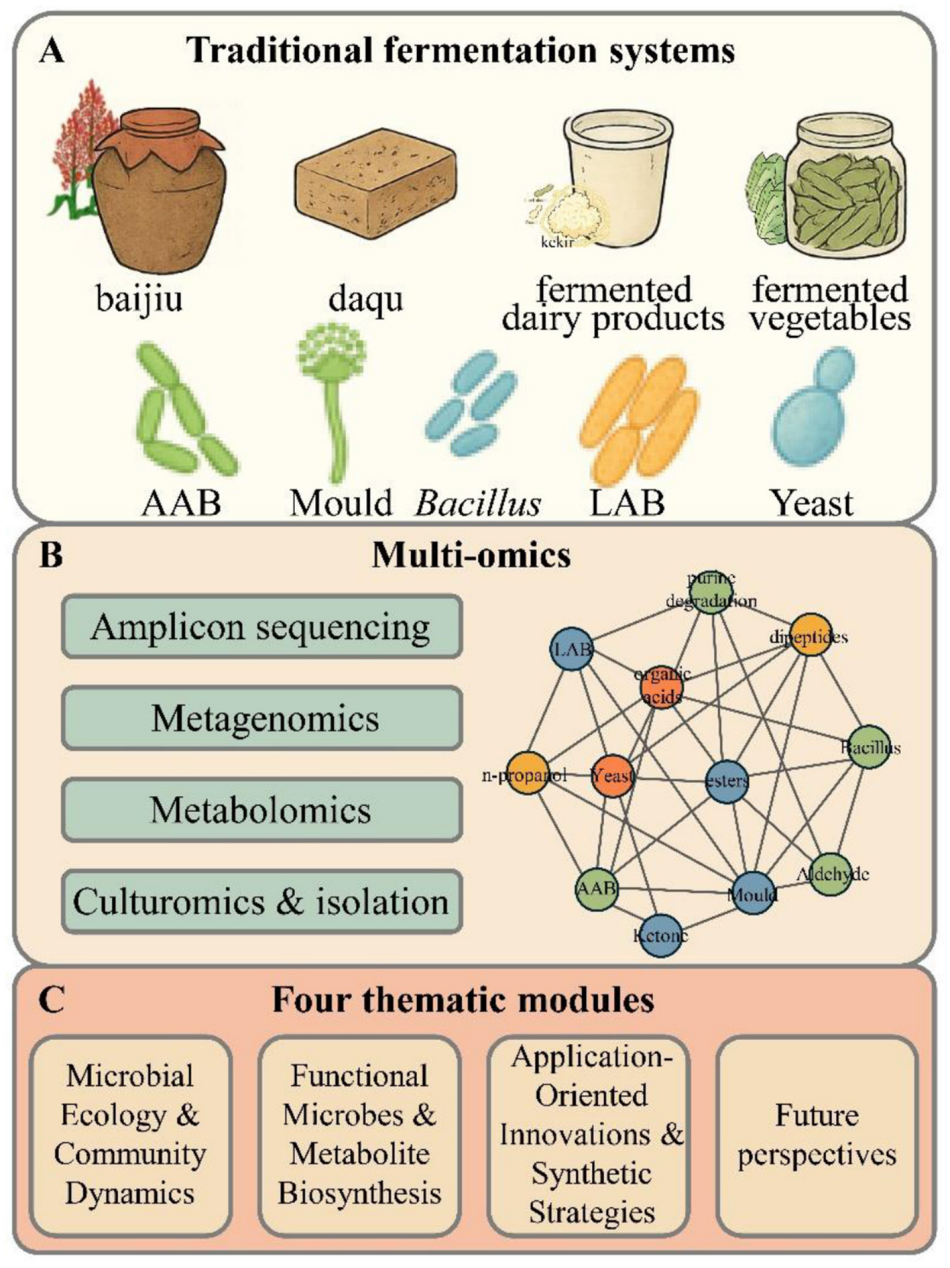
Overview of the diversity, analytical approaches, and thematic structure of traditional food fermentation research. **(A)** Representative traditional fermentation systems and their core microbial groups. **(B)** Multi-omics approaches for resolving community structure and function. **(C)** Four thematic modules synthesized in this Research Topic.

## Microbial ecology and community dynamics

Several studies in this Research Topic leverage advanced sequencing technologies to systematically characterize the microbial ecology of traditional fermented foods. Sanz-López et al. combined culture-dependent methods with amplicon sequencing to investigate the microecology of several Ethiopian traditional fermented products, including two foods (Kotcho and Injera), one condiment (Datta), and six beverages (Tej, Tella, Cheka, Kinito, Borde, and Shamita). Their analyses revealed diverse bacterial communities in which *Acetobacter, Lactiplantibacillus, Lentilactobacillus*, and *Paenibacillus* were among the highly abundant taxa. Similarly, Shen et al. used metagenomics to compare the microbiomes of Jiang-flavor Baijiu fermented grains from different regions, characterizing community structures and core functional genes while offering recommendations for standardizing Daqu management. Xie et al. focused on a traditional Chinese fermented vegetable (bangcai), integrating GC-IMS with 16S/ITS sequencing to demonstrate a strong correspondence between microbial succession and the development of volatile flavors. *Citrobacter, Lactobacillus*, and *Leuconostoc* were identified as dominant genera tightly linked to the production of fruity and floral compounds. Wang et al. explored the microbiome of an herbal processing fermentation: the “stack sweating” of *Eucommiae Cortex* (a medicinal bark). They observed significant shifts in bacterial community (mainly *Gluconobacter*, unclassified_c_*Gammaproteobacteria, Pseudomonas, Pantoea, Pedobacter*, and *Parecoccus*) during the sweating process, which correlated with increases in key bioactive compounds (alkaloids, amino acid related compounds, flavonoids, phenylpropanoids and terpenoids). Their network analysis linked core microbes to metabolite pathways, providing a scientific basis for optimizing this traditional herbal preparation. Collectively, these studies demonstrate that high-resolution analyses of community dynamics offer powerful insights into the microbial mechanisms shaping sensory qualities in traditional fermentations.

Within Baijiu fermentation systems, Lin et al. analyzed the fermented grains of light-aroma Baijiu and identified a clear temporal division of labor between abundant and rare taxa. Although dominant species declined in diversity during late fermentation, low-abundance “rare” taxa increased in diversity and emerged as keystone nodes in the microbial network. These findings highlight that rare microbes, often overlooked, may play essential roles in maintaining community stability and functional resilience. In high-temperature Daqu, Xu et al. introduced a defined synthetic functional microflora that substantially reshaping community structure and enzymatic profiles. Inoculation with selected yeasts and molds enhanced saccharification, fermentation, and esterification capacities, increased the abundance of beneficial *Bacillus*, and suppressed certain *Lactobacillus* and *Weissella*, thereby steering community assembly along a more desirable trajectory. Under this controlled bioturbation framework, Daqu quality improved in a consistent and reproducible manner. Meanwhile, Mandlaa et al. focused on acetic acid bacteria in strong-flavor Baijiu, mapping their distribution across fermentation stages and locations and underscoring their central contribution to acetic acid and ester formation.

Microbial diversity has also been a central theme in studies of traditional fermented dairy and cereal products. Mudoor Sooresh et al. showed that freeze-dried kefir starter cultures prepared from authentic kefir grains can successfully recapitulate the microbial community and flavor of fresh kefir, offering practical value for the development of commercially scalable starter cultures. Su et al. compared 307 *Saccharomyces cerevisiae* strains derived from two contrasting fermentation sources, namely winemaking and Chinese Mantou fermentation, and profiled their divergent stress-adaptation traits. This provides a compelling exploration of *S. cerevisiae* domestication. Oviedo-Hernández et al. reassessed intraspecies genetic diversity patterns in *Oenococcus oeni*, the key bacterium responsible for malolactic fermentation in wine, and uncovered population structures that challenge traditional strain classifications.

Collectively, the diversity-focused cluster underscores the unique value of traditional fermentations as natural laboratories for microbial ecology. By resolving community structures and dynamics, these studies illuminate the ecological principles underlying fermentation quality and pinpoint key taxa and interactions suitable for targeted application.

## Functional microbes and metabolite biosynthesis

This thematic cluster centers on core microbes that carry out key biochemical functions and thereby shape flavor or health attributes, together with the biosynthesis of their metabolites. The first thread concerns microbial origins and mechanisms of aroma production and flavor precursors. Li et al. reported that a plant endophytic bacterium, *Bacillus velezensis*, can produce cyclic dipeptides at high levels. Upon thermal cracking, these compounds release key nutty, roasted, and fruity notes, and their application significantly enhances product flavor. Zhang et al. demonstrated that *Lentilactobacillus diolivorans* strain ZX6 generates abundant n-propanol in sauce-aroma Baijiu via the methylglyoxal pathway, that is, proceeding from pyruvate or lactate to 1,2-propanediol and then to propanol, catalyzed by aldehyde dehydrogenase. Quantitative PCR results showed its widespread presence in fermentation pits, identifying it as an important source of higher alcohols. This suggests that monitoring and regulating this bacterium could be leveraged to manage n-propanol levels and flavor balance. Granchi et al. compared sequential inoculation strategies of *Metschnikowia pulcherrima* and *Torulaspora delbrueckii* with *S. cerevisiae* and found that both non-*Saccharomyces* yeasts enhanced aroma intensity and complexity. The former increased esters, while the latter elevated glycerol and polysaccharides and improved mouthfeel. This validates the effectiveness of multi-yeast synergy for broadening flavor spectra.

The second thread concerns microbial sources and evaluation of health-functional metabolites and activities. A relatively complete evidential chain has now formed around systematic screening of lactic acid bacteria for health-related enzymatic activities, spanning *in vitro* enzymatic inhibition and substrate degradation to *in vivo* efficacy and safety verification. Liu et al. isolated purine-degrading lactic acid bacteria from traditional foods of Yunnan, including *Limosilactobacillus fermentum* and *Pediococcus acidilactici*. Among them, *L. fermentum* MX-7 reduced serum uric acid and improved renal indicators in a rat model, with efficacy comparable to allopurinol. Liu et al. discovered that 10 out of 27 lactic acid bacteria strains from Guizhou fermented foods exhibited alpha-glucosidase inhibitory activity at 30–39%, mainly belonging to *Weissella* and *Lactobacillus*, and these strains showed acid and bile tolerance. Gui et al. isolated *Lactobacillus paracasei* from Jiangshui and co-fermented it with yeast in Jerusalem artichoke juice. The resulting beverage achieved an alpha-glucosidase inhibition rate of 83% and markedly enhanced antioxidant capacity. Meanwhile, *Schleiferilactobacillus harbinensis* reported by Liu et al. performed well at pH 3.5 with 0.3% bile salts, exhibited antibacterial activity, and increased free amino acids during cereal fermentation. *Weissella confusa* SY628 reported by Liu et al., after systematic safety evaluation, was used for co-fermentation of soy yogurt, significantly improving texture and flavor. At a pH around 4.5, it maintained more than 5 × 10^7^ viable cells per milliliter (CFU/mL) during 21 days of refrigerated storage, which reflects excellent shelf-life and product stability. Overall, these studies translate health goals such as purine degradation, glycemic control, and antioxidant activity into reproducible strains and processes, and they establish executable evaluation workflows and formulation routes.

Taken together, the functional microbes cluster clearly showcases two parallel value chains. The first involves flavor enhancement driven by specialized metabolites and enzymatic activities. The second involves health promotion mediated by probiotic functions and targeted biotransformation. By establishing reproducible links between specific microbes and defined metabolites or physiological effects, these studies make a transition from descriptive ecology to functional food microbiology and provide industry with directly verifiable, scalable strain candidates and implementation roadmaps.

## Application-oriented innovations and synthetic strategies

Building on advances in understanding microbial diversity and function, several studies shift toward process innovation and application-focused development, with an emphasis on steering fermentation outcomes through synthetic or well-defined microbial consortia. Gao et al. engineering a synthetic microbial community, SMC-L1, for low-salt soy sauce. Traditionally, foy sauce required ~18% NaCl for microbial control, but by combining *Tetragenococcus halophilus*, a halophilic lactic acid bacterium, with compatible yeasts, and using multi-omics optimization, they achieved stable fermentation at just 13% salt. The resulting soy sauce contained ~40% more amino nitrogen than the control and exhibited richer flavor metabolites, including elevated succinate that was driven by a *Tetragenococcus*-mediated anaerobic TCA cycle and short-chain esters. This work effectively decoupled salinity from flavor quality and offered a promising route toward healthier condiments. Similarly, OuYang et al. assembled a defined consortium from metabolically complementary strains, which significantly increased acetoin content and improved overall functional quality in citrus vinegar. Through targeted metabolic optimization, the consortium outperformed traditional monoculture conditions, highlighting the potential of rationally designed communities to enhance food fermentation process.

In traditional fermented dairy products and beverages, advances in starter-culture technology are equally notable. Mudoor Sooresh et al. tackled the challenge of preserving the complex microbiota of kefir grains. They showed that Freeze-dried starters derived from authentic grains could inoculate milk to produce products nearly indistinguishable from fresh kefir in both flavor and community structure, which provides a practical solution for standardized production in regions without access to live grains. Chen et al. extended kefir fermentation to a plant-based matrix. During 4 months of serial transfer in soymilk, the kefir community underwent adaptive reshaping. *Lactobacillus kefiranofaciens*, initially dominant ~95% in dairy kefir, dropped to 16%, while *Lacticaseibacillus paracasei*, capable of metabolizing soymilk oligosaccharides such as raffinose and stachyose, rose to ~77% and became the dominant species. This shift reduced kefiran production, grain size, and texture quality and led to declines in sensory attributes and ACE inhibitory activity. Even so, the study establishes a foundation for plant-based kefir development and suggests strategies to maintain functional robustness, such as periodic rejuvenation in dairy matrices or supplementation with exopolysaccharide-producing strains.

From a broader perspective, Su et al. demonstrated the potential of cross-scenario strain utilization. Yeasts originating from Chinese *mantou* fermentation differ markedly from wine yeasts in stress tolerance, which reflects distinct domestication histories. Leveraging such adaptive features may enable targeted selection for conditions such as high sugar, high osmolarity, elevated ethanol, or low pH.

Overall, traditional foods worldwide provide a broad strain and process library for application-oriented innovation. Ethiopian fermented foods rich in *Bacillus* and probiotic-potential lactic acid bacteria, Chinese fermented vegetables with enzyme-inhibitory activities, and Baijiu fermented grains yielding novel yeast and mold isolates can all serve as a source pool for next-generation starters and functional food components.

## Future perspectives for research and industry

Looking forward, traditional food fermentation must move from experience-driven practice toward predictability and verifiable design. Integrating amplicon, metagenomic, and metabolomic data with kinetic models offers a pathway to constructing simplified digital-twin fermentation systems. Machine learning and other artificial intelligence approaches can further enable pre-screening of community formulations and process parameters before bench-scale experimentation and scale-up. Central to this shift is the use of dose-response relationships that map strain to metabolites and, ultimately, to flavor or functional outcomes, thereby guiding rational parameter selection. Such approaches can reduce trial and error, shorten development timelines, and ensure product quality.

Reproducibility begins with safeguarding microbial diversity. We recommend establishing microbial resource banks that cover traditional starters such as Daqu, koji, sourdough, and kefir, along with standardized protocols for isolation, preservation, revival, and activation, in order to prevent losses of flavor and stability that can accompany industrial simplification. In parallel, practices such as back-slopping and raw-material pretreatment should be documented, with sources and authorizations clearly specified, to form a traceable body of process knowledge.

Functional claims must be supported by clear lines of evidence. Begin with *in vitro* assays to confirm enzymatic inhibition or substrate degradation. Then quantify dose–response relationships with sensory and physicochemical indices in food models. Finally, verify efficacy and safety in animal or human studies. By combining multi-omics with metabolic flux analysis, one can identify which classes of metabolites attain effective concentrations in actual foods and, on this basis, define the minimum effective intake. These data directly inform label compliance and regulatory submissions.

On the application front, priority can be given to low-salt, low-sugar, and plant-based scenarios. By combining substrate adaptation, community remodeling, and supplementation with exogenous metabolites, texture and flavor can be improved. For example, in plant-based fermentations, supplementing exopolysaccharide-producing strains or adding flavor precursors can counterbalance deficits in mouthfeel and aroma. Finally, by unifying data recording formats for multi-omics, process parameters, and sensory results, we can construct shareable evaluation datasets that facilitate reproducibility and benchmarking across facilities.

In conclusion, this Research Topic systematically elucidates how microbial diversity can be translated into measurable, controllable functions, and further clarifies how these functions can be harnessed through targeted process innovation. Collectively, the studies presented here contribute to a more mature and integrated conceptual framework. Traditional fermentation should be viewed not only as a cultural heritage to be preserved, but also as dynamic frontiers driving scientific discovery and biotechnological progress. Through in-depth profiling and comparative analyses of fermentation microbiomes across diverse regions and raw-material contexts, we can generate actionable strategies for improving product quality, enhancing nutritional value, and advancing sustainability. Such approaches ensure that the microbial resources and functional potential embedded in traditional fermented foods continue to deliver stable, reliable, and health-and-flavor-aligned societal benefits at scale.

